# Improved glycemic regulation on exercise compared to non‐exercise days in a real‐world setting in individuals recently diagnosed with type 2 diabetes: A secondary analysis of the MOTIVATE T2D randomised controlled trial

**DOI:** 10.1111/dom.16623

**Published:** 2025-07-21

**Authors:** Jonathan L. Low, Katie Hesketh, Kaja Falkenhain, Mary E. Jung, Joel Singer, Charlotte A. Jones, Catherine Russon, Rob Andrews, Matthew Cocks, Ali M. McManus, Jonathan P. Little, the MOTIVATE T2D Team

**Affiliations:** ^1^ School of Health and Exercise Sciences University of British Columbia Kelowna British Columbia Canada; ^2^ Research Institute for Sport and Exercise Sciences Liverpool John Moores University Liverpool UK; ^3^ School of Sport, Exercise and Rehabilitation Sciences University of Birmingham Birmingham UK; ^4^ Pennington Biomedical Research Center Louisiana State University Baton Rouge Louisiana USA; ^5^ School of Population and Public Health University of British Columbia Vancouver British Columbia Canada; ^6^ Faculty of Medicine University of British Columbia Kelowna British Columbia Canada; ^7^ University of Exeter Medical School University of Exeter Exeter UK

**Keywords:** acute exercise, continuous glucose monitoring, diabetes, glycemic regulation, mHealth, real‐world, type 2 diabetes

## 1 INTRODUCTION

Most studies on exercise and glycemic regulation have been conducted in supervised laboratory/research settings where exercise compliance is ensured with diet and medications controlled^1^, ^2^, ^3^, ^4^, ^5^ making it difficult to draw real‐world insights. New developments in technology have made real‐world monitoring of exercise a viable alternative to traditional in‐person exercise supervision.^6^ However, the effects of free‐living exercise on glycemic regulation are virtually unknown. We aimed to determine if a ≥10‐min bout of purposeful exercise performed in a real‐world setting leads to improvements in glucose regulation assessed by continuous glucose monitoring (CGM) in individuals with recently diagnosed type 2 diabetes.

## 2 METHODS

### 2.1 Research design

We performed a secondary analysis of the MOTIVATE T2D trial (NCT04653532), a two‐centre (UK and Canada), parallel‐group randomized controlled trial (RCT) investigating the feasibility of an mHealth exercise intervention vs. active control for individuals with recently (5–24 months) diagnosed type 2 diabetes. Detailed methods are provided in the published protocol^7^ and main trial papers.^8^ Key eligibility criteria can be found in Table S1 and Supporting Information Methods.

Participants (*N* = 120) completed baseline assessments before a 26‐week exercise intervention. Follow‐up assessments took place during the final 2 weeks of the intervention (sample time period 1, T1; weeks 24–26) and 6 months later (sample time period 2, T2; weeks 50–52). During both 14‐day periods, participants wore a blinded continuous glucose monitor (Freestyle Libre Pro, Abbott Technologies) on the upper arm while continuing their exercise programme. Exercise sessions in the intervention group were tracked using a Polar Ignite fitness watch and Polar Verity Sense heart rate strap. As heart rate data were required to verify exercise sessions, data from the intervention group (*n* = 61) only were analysed.

### 2.2 Exercise program

Over 24 weeks leading to T1, participants received a personalized progressive exercise program (performed independently). The exercise prescription aimed to increase intensity and duration in the first 12 weeks to meet the physical activity (PA) guidelines and then maintain this level of activity for the next 14 weeks (150–300 min of moderate‐to‐vigorous activity per week).^5^

#### 2.2.1 Progression of Exercise Program

Although individualized, exercise progression was guided by an age‐predicted maximum heart rate (HR_max_)‐based framework:

1. **Moderate aerobic training** began at 50%–60% HR_max_ for 20 min per session in week 1 and incrementally increased to 45 min by week 12, maintaining the same heart rate (HR) range throughout. Warm‐up included an additional 5 min at low intensity (<60% HR_max_).
2. **Vigorous aerobic training** also started at 50%–60% HR_max_—6 min at the HR_max_ target in week 1—with session duration rising to 25 min at the HR_max_ target by week 12. In week 3, the HR_max_ target shifted upward to 60%–70% for the remainder of the intervention. Warm‐up included an additional 5 min at low intensity (<60% HR_max_).
3. **Interval training** was prescribed at 80%–90% HR_max_, with total work time progressing from 4 min at the HR_max_ target in week 1 to 9 min at the HR_max_ target by week 12. Warm‐up included an additional 2 min at low intensity (<60% HR_max_).
4. **Strength training** started with three sets of five exercises per session and was scaled to four sets of six exercises by week 12. Warm‐up included an additional 2 min at low intensity (<60% HR_max_).

Exercises were individually tailored and included moderate and vigorous aerobic, interval, and strength training based on participant preference. All exercise types (aerobic, strength, classes, sports) and modes (gym, outdoor, home, commuting) were offered to increase adherence. From weeks 27–52, participants maintained their exercise routine with no further support.

### 2.3 Exercise session analysis

Raw 1‐Hz HR data were extracted for each exercise session and compared with time‐aligned CGM data from T1 (24–26 weeks with support; main analyses) and T2 (50–52 weeks without support; supplementary analyses).

Sessions ≥10 min in duration were included and corroborated by cadence/speed/GPS data, participant notes, and text messages. Valid sessions contributed to the analysis of exercise and non‐exercise days. An exercise day was defined as the 24‐h period following an exercise session. A non‐exercise day was a 24‐h period without exercise in the preceding 48 h, in line with guidelines that state no more than 2 days should elapse between exercise sessions.^5^

### 2.4 CGM analysis

CGM data were retrieved using LibreView software. Time‐aligned exercise and non‐exercise days were marked in the CGM file, and metrics were calculated for the 24 h after using the Diametrics platform.^9^ Sessions required valid HR data, a CGM reading within 10 min before the exercise session, and consistent CGM data for 24 h afterward.

### 2.5 Statistical analysis

Baseline characteristics are presented as means (standard deviation, SD) for continuous or *N* (%) for categorical variables. A linear mixed model examined differences in the primary (24‐h mean glucose) and secondary (other CGM metrics) outcomes between exercise and non‐exercise days at T1 and T2. Further details can be found in Supporting Information Methods.

## 3 RESULTS

### 3.1 Study participants

At T1, 45/61 participants (49% female) had sufficient CGM data for at least one exercise session/day in the 14‐day period. Participant characteristics are provided in Table 1.

**TABLE 1:** Descriptive statistics of participants included in analyses at the primary 24‐week time point (T1).

Descriptive characteristic	Participant characteristic
	Time point 1 (T1)
Overall, *N*	45
Sex	—
Male, *n* (%)	23 (51)
Female, *n* (%)	22 (49)
Age, years, mean (SD)	57 (8)
40–60, *n* (%)	30 (67)
>60, *n* (%)	15 (33)
Diagnosis, months	13 (7)
0–6, *n* (%)	13 (29)
7–12, *n* (%)	11 (24)
13–18, *n* (%)	10 (22)
19–24, *n* (%)	11 (24)
Baseline HbA1c, %, mean (SD); mmol/mol, mean (SD)	6.7 (1.1); 50 (12.0)
<6.0, *n* (%)	10 (22)
6.0–6.5, *n* (%)	11 (24)
6.5–7.0, *n* (%)	10 (22)
7.0–7.5, *n* (%)	4 (9)
7.5–8.0, *n* (%)	2 (4)
>8.0, *n* (%)	4 (9)
BMI (body mass index), kg/m^2^, mean (SD)	32.9 (5.3)
<25.0, *n* (%)	1 (2)
25.0–30.0, *n* (%)	12 (27)
>30.0, *n* (%)	32 (71)
Mean arterial pressure, mmHg, mean (SD)	95.6 (9.6)
<90, *n* (%)	11 (24)
90–92, *n* (%)	8 (18)
92–96, *n* (%)	3 (7)
>96, *n* (%)	23 (51)
CGM wear time, days, mean (SD)	12.6 (2.5)
<7, *n* (%)	3 (7)
7–10, *n* (%)	1 (2)
11–14, *n* (%)	41 (91)
Total exercise‐energy expenditure during 2 weeks, kcal, mean (SD)	1757 (1501)
<500, *n* (%)	7 (16)
500–1500, *n* (%)	20 (44)
>1500, *n* (%)	18 (40)
Number of exercise sessions over the 2 weeks, mean (SD)	7 (7)
0, *n* (%)	0 (0)
1, *n* (%)	6 (13)
2, *n* (%)	5 (11)
3, *n* (%)	3 (7)
>3, *n* (%)	31 (69)

*Note*: Descriptive statistics of the participants at T1.

### 3.2 Exercise sessions and heart rate

Three‐hundred exercise sessions were recorded from 45 participants at T1 and 105 sessions were recorded among 18 participants at T2. HR data and a breakdown of exercise types comprising the exercise sessions can be found in Table S8. Sixty‐three percent of the exercise days were measured on a weekday and 37% were on a weekend. Additional characteristics of the participants contributing exercise sessions to the analysis at T1 are in Table 2 (see Table S3 for T2 details).

**TABLE 2:** Descriptive statistics of the exercise sessions included in analyses at the primary 24‐week time point (T1).

Descriptive characteristic	Exercise sessions characteristic
	Time point 1 (T1)
Overall, *N*	300
Sex	—
Male, *n* (%)	189 (63)
Female, *n* (%)	111 (37)
Age, years, mean (SD)	—
40–60, *n* (%)	187 (62)
>60, *n* (%)	113 (38)
Diagnosis, months	—
0–6, *n* (%)	86 (29)
7–12, *n* (%)	57 (19)
13–18, *n* (%)	59 (20)
19–24, *n* (%)	98 (33)
Baseline HbA1c, %, mean (SD); mmol/mol, mean (SD)	—
<6.0, *n* (%)	65 (22)
6.0–6.5, *n* (%)	132 (44)
6.5–7.0, *n* (%)	50 (17)
7.0–7.5, *n* (%)	15 (5)
7.5–8.0, *n* (%)	6 (2)
>8.0, *n* (%)	17 (6)
BMI (body mass index), kg/m^2^, mean (SD)	—
< 25.0, *n* (%)	10 (3)
25.0–30.0, *n* (%)	92 (31)
>30.0, *n* (%)	198 (66)
Mean arterial pressure, mmHg, mean (SD)	—
<90, *n* (%)	53 (18)
90–92, *n* (%)	35 (12)
92–96, *n* (%)	21 (7)
>96, *n* (%)	191 (64)
Exercise session duration, minutes, mean (SD)	65 (43)
<10, *n* (%)	0 (0)
10–30, *n* (%)	78 (26)
>30, *n* (%)	221 (74)
Average HR, bpm, mean (SD)	110 (9)
<100 bpm, *n* (%)	61 (21)
100–120 bpm, *n* (%)	170 (60)
>120 bpm, *n* (%)	53 (19)

*Note*: Descriptive statistics of the exercise sessions at T1.

### 3.3 Non‐exercise days

A total of 106 non‐exercise days were identified at T1 and 53 were identified at T2. Fifty‐six percent of the non‐exercise days were measured on a weekday and 44% were on a weekend.

### 3.4 Glycemic regulation on exercise versus non‐exercise days

**FIGURE 1:**
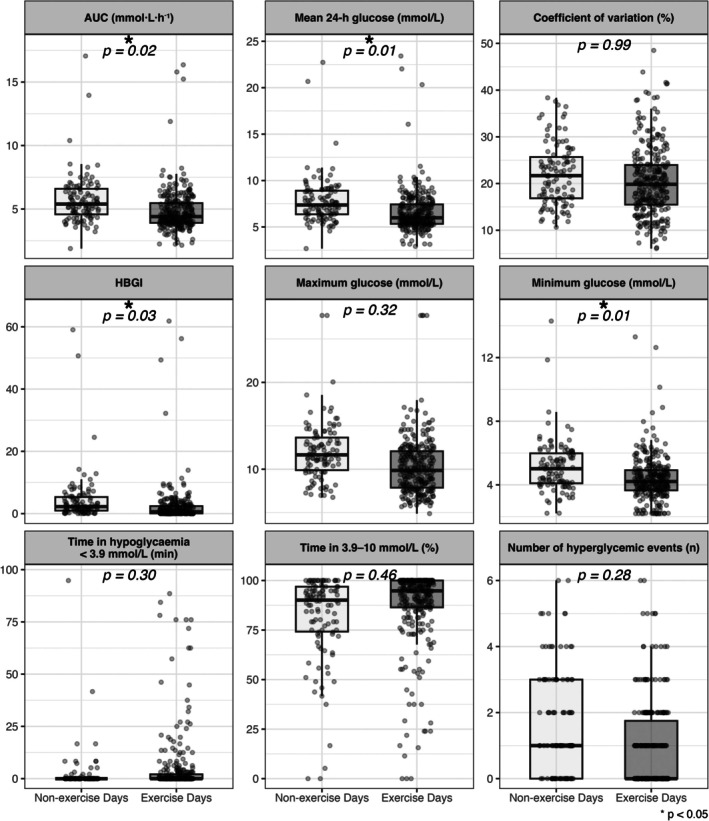
Estimated marginal means with individual data points and the corresponding confidence intervals from continuous glucose monitoring (CGM) outcome variables at the 24‐week time point (T1) based on *N* = 45 participants with valid CGM data and at least one exercise session. * indicates statistical significance, *p < 0.05*.

At T1, a significant reduction of −0.2 mmol/L (95% CI [−0.4, 0.0], *p* = 0.01) in 24‐h mean glucose was evident when comparing exercise days to non‐exercise days (Figure 1). Compared with non‐exercise days, exercise days also had lower minimum glucose (−0.3 mmol/L; 95% CI [−0.5, −0.1], *p* = 0.01), lower area under the curve (AUC) (−0.2 mmol/L/h.; 95% CI [−0.3, −0.9], *p* = 0.03), lower high blood glucose index (HBGI; −0.6; 95% CI [−1.2, −0.1], *p* = 0.03) (Figure 1), and a reduction in time spent in level 2 hyperglycemia (above 13.9 mmol/L) (−1.2%; 95% CI [−2.4, −0.1], *p* = 0.03) (Table S4). These results are supported by analysis of the exercise sessions from T2, where significant reductions in mean 24‐h glucose, minimum glucose, AUC, and HBGI were also noted (see Table S6). For completeness, all consensus reporting CGM metrics^10^ can be found in Tables S6 and S7.

## 4 DISCUSSION

This study provides the first, to our knowledge, real‐world evidence that individuals living with recently diagnosed type 2 diabetes have improved glycemic regulation for 24 h after a bout of unsupervised exercise.

The primary strength of this study is that it presents results from a large sample of free‐living exercise sessions, with high ecological validity, showing that exercise can benefit glycemic regulation outside of controlled lab‐based studies. Knowing that a bout of exercise, performed in the real world without supervision or dietary control, can improve CGM metrics, should empower patients and clinicians. A reduction in mean 24‐h glucose, minimum glucose, glucose AUC, and HBGI would be expected over time to contribute to improvements in clinical markers such as glycated haemoglobin (HbA1C); supported by the improved estimated glycated haemoglobin (eA1C) in our results (Table S3). The synergy arising from the mHealth nature of the study also highlights the potential for this approach to exercise therapy when hands‐on resources are limited.

Several factors warrant consideration when interpreting these findings. This study was a secondary, exploratory analysis of a larger RCT, and data were not collected with the primary aim of addressing the research question reported here (e.g., an a priori sample size calculation was not conducted). Only 45 participants (300 exercise vs. 106 non‐exercise days) contributed CGM data at post‐intervention and 18 participants at 6‐month follow‐up (105 exercise vs. 53 non‐exercise days), so though the day‐level mixed‐effects estimates may be strong at T1 and exploratory at T2, both require confirmation in larger cohorts. Secondly, the glucose‐lowering effect of real‐world exercise appeared lower than prior studies with supervised exercise and controlled dietary conditions, which is perhaps not surprising given that exercise dose, diet, and medications were not standardized.^1^, ^2^, ^4^ Although this analysis lacked dietary, pharmacologic, or behavioural standardization (e.g., medication adjustments, sleep, or stress), this “uncontrolled” context is also a key strength. It demonstrates that even in the real world, bouts of unsupervised exercise yield modest but clinically meaningful improvements in 24‐h glycemic control. The mechanisms underlying lower 24‐h glucose following exercise are likely related to improved insulin sensitivity, but we cannot rule out other potential contributing factors (e.g., compensatory changes to diet, sleep, stress, activity, etc.). Additionally, individual variability in response to exercise and underlying metabolic health may modulate the magnitude of glycemic changes observed, and the study was not able to explore individual predictors of response.

In conclusion, we provide the first real‐world evidence that a single bout of exercise lowers glucose over the subsequent 24 h in people with recently diagnosed type 2 diabetes. The findings explicitly reinforce the PA guidelines for type 2 diabetes, which state that individuals should not allow more than 2 days to elapse between exercise sessions.^11^ This study adds important real‐world data to support the role of exercise in managing blood glucose in people with type 2 diabetes.

## AUTHOR CONTRIBUTIONS

J.L.L., K.H., M.C., A.M.M., M.E.J., and J.P.L. were involved in the conception, design, and conduct of the study, as well as the analysis and interpretation of the results. K.F., C.R. and J.S. assisted in statistical and data analysis. J.L.L. wrote the first draft of the manuscript, and all authors edited, reviewed, and approved the final version of the manuscript. J.P.L. is the guarantor of this work and, as such, had full access to all the data in the study and takes responsibility for the integrity of the data and the accuracy of the data analysis.

## FUNDING INFORMATION

This study was supported by a UK‐Canada Diabetes Research Team Grant from the Medical Research Council (MRC) and Canadian Institutes of Health Research (CIHR). J.P.L. is University of British Columbia Okanagan Principal's Research Chair in Metabolism.

## CONFLICT OF INTEREST STATEMENT

The authors declare no conflicts of interest.

## PEER REVIEW

The peer review history for this article is available at https://www.webofscience.com/api/gateway/wos/peer‐review/10.1111/dom.16623.

## Supporting information


**Data S1.** Supporting Information.

## Data Availability

The de‐identified participant‐level data that support the findings of this study are available from the corresponding author upon reasonable request. Aggregate summary data are provided in the published article and its Supporting Information.
