# Supplementary material for: Improved glycemic regulation on exercise compared to non‐exercise days in a real‐world setting in individuals recently diagnosed with type 2 diabetes: A secondary analysis of the MOTIVATE T2D randomised controlled trial
Source: Diabetes Obes Metab. 2025 Jul 21;27(10):6044–9. doi: 10.1111/dom.16623 (PMC12409211; doi:10.1111/dom.16623)
Supplement: Supplementary file 1 — Data S1. Supporting Information. [file DOM-27-6044-s001.docx]

**SUPPLEMENTAL MATERIAL**

**TABLES**

**Supplemental Table 1.** Key Eligibility Criteria for the MOTIVATE T2D trial

| **Inclusion Criteria** | **Exclusion Criteria** |
| --- | --- |
| 1. *Recent clinical diagnosis of type 2 diabetes (5-24 months)* 2. *Age 40-75 years* 3. *Managing type 2 diabetes by lifestyle or metformin only (stable dose ≥3 months)* 4. *No regular exercise (<150 min/week)* | 1. *Age <40 or >75 years* 2. *HbA1c > 10% (86 mmol/mol)* 3. *Blood pressure > 160/100 mm Hg* 4. *Managing type 2 diabetes with antidiabetic medications other than metformin* 5. *History of cardiovascular events within last 3 months* 6. *Pregnant or <6 months postpartum* 7. *Those without a smartphone or data/WIFI access* |

Key inclusion and exclusion criteria for the study. Further descriptions can be found in Supplemental Methods.

**Supplemental Table 2.** Descriptive Statistics of Participants Included in Analyses at T2

| Descriptive characteristic | Participant Characteristics |
| --- | --- |
|  | Time Point 2 |
| Overall, N | 18 |
| Sex | - |
| Male, n (%) | 12 (67) |
| Female, n (%) | 6 (33) |
| Age, years, mean (SD) | 56 (7) |
| 40-60, n (%) | 13 (72) |
| >60, n (%) | 5 (28) |
| Diagnosis, months | 13 (7) |
| 0-6, n (%) | 7 (39) |
| 7-12, n (%) | 2 (11) |
| 13-18, n (%) | 4 (22) |
| 19-24, n (%) | 5 (28) |
| HbA1c, %, mean (SD) | 6.6 (1.6) |
| <6.0, n (%) | 7 (39) |
| 6.0-6.5, n (%) | 5 (28) |
| 6.5-7.0, n (%) | 2 (11) |
| 7.0-7.5, n (%) | 2 (11) |
| 7.5-8.0, n (%) | 0 (0) |
| >8.0, n (%) | 1 (6) |
| BMI, kg/m^2^, mean (SD) | 32.8 (4.4) |
| <25.0, n (%) | 0 (0) |
| 25.0-30.0, n (%) | 4 (22) |
| >30.0, n (%) | 14 (78) |
| Mean arterial pressure, mmHg, mean (SD) | 93.1 (8.8) |
| <90, n (%) | 7 (39) |
| 90-92, n (%) | 1 (6) |
| 92-96, n (%) | 2 (11) |
| >96, n (%) | 8 (44) |
| CGM wear time, days, mean (SD) | 12.8 (1.8) |
| < 7, n (%) | 0 (0) |
| 7-10, n (%) | 2 (11) |
| 11-14, n (%) | 16 (89) |
| TIR 70-180, mg/dL 24 hr before exercise, % | 82 (26) |
| <60 | 2 (11) |
| 60-80 | 0 () |
| >80 | 14 (78) |
| Time <70 mg/dL 24 hr before exercise, % | 4 (6) |
| <1 | 10 (56) |
| 1-5 | 2 (11) |
| >5 | 4 (22) |
| Total exercise-energy expenditure during 2 weeks, kcal, mean (SD) | 2314 (2478) |
| <500, n (%) | 4 (22) |
| 500-1500, n (%) | 5 (28) |
| >1500, n (%) | 9 (50) |
| Number of exercise sessions over the 2 weeks, mean (SD) | 6 (5) |
| 0, n (%) | 0 (0) |
| 1, n (%) | 3 (17) |
| 2, n (%) | 3 (17) |
| 3, n (%) | 2 (11) |
| >3, n (%) | 10 (56) |

Descriptive statistics of the participants at T2.

**Supplemental Table 3.** Descriptive Statistics of the Exercise Sessions Included in Analyses at the T2

| Descriptive characteristic | Exercise Sessions Characteristics |
| --- | --- |
|  | Time Point 2 |
| Overall, N | 105 |
| Sex | - |
| Male, n (%) | 82 (78) |
| Female, n (%) | 23 (22) |
| Age, years, mean (SD) | - |
| 40-60, n (%) | 73 (70) |
| >60, n (%) | 32 (30) |
| Diagnosis, months | - |
| 0-6, n (%) | 39 (37) |
| 7-12, n (%) | 6 (6) |
| 13-18, n (%) | 21 (20) |
| 19-24, n (%) | 39 (37) |
| HbA1c, %, mean (SD) | - |
| <6.0, n (%) | 54 (51) |
| 6.0-6.5, n (%) | 21 (20) |
| 6.5-7.0, n (%) | 16 (15) |
| 7.0-7.5, n (%) | 11 (10) |
| 7.5-8.0, n (%) | 0 (0) |
| >8.0, n (%) | 2 (2) |
| BMI, kg/m^2^, mean (SD) | - |
| <25.0, n (%) | 0 (0) |
| 25.0-30.0, n (%) | 13 (12) |
| >30.0, n (%) | 92 (88) |
| Mean arterial pressure, mmHg, mean (SD) | - |
| <90, n (%) | 35 (33) |
| 90-92, n (%) | 2 (2) |
| 92-96, n (%) | 30 (29) |
| >96, n (%) | 38 (36) |
| TIR 70-180, mg/dL 24 hr before exercise, % | - |
| <60 | 48 (46) |
| 60-80 | 8 (8) |
| >80 | 38 (36) |
| Time <70 mg/dL 24 hr before exercise, % | - |
| <1 | 70 (67) |
| 1-5 | 14 (13) |
| >5 | 10 (10) |
| Exercise session duration, minutes, mean (SD) | 88 (88) |
| <10, n (%) | 0 (0) |
| 10-30, n (%) | 20 (19) |
| >30, n (%) | 85 (81) |
| Average HR, bpm, mean (SD) | 111 (9) |
| < 100 bpm, n (%) | 19 (20) |
| 100-120 bpm, n (%) | 56 (58) |
| > 120 bpm, n (%) | 22 (23) |

Descriptive statistics of the exercise sessions at T2.

**Supplemental Table 4.** Additional CGM Metrics at T1.

| Metric | Exercise Day | No Exercise Day | Between-Group Effect Estimate |
| --- | --- | --- | --- |
| Mean 24-hour glucose, mmol/L | 6.9 (6.6, 7.3) | 7.1 (6.8, 7.5) | -0.2 (-0.4, -0.04), *p* = 0.01 |
| SD, mg/dL | 27.1 (24.1, 30.1) | 27.9 (24.8, 31.1) | -0.8 (-2.6, 0.9), *p* = 0.36 |
| SD, mmol/L | 1.5 (1.3, 1.7) | 1.6 (1.4, 1.7) | -0.1 (-0.1, 0.1), *p* = 0.36 |
| eA1c, % | 5.9 (5.8, 6.2) | 6.1 (5.9, 6.4) | -0.2 (-0.3, -0.03), *p* = 0.01 |
| Minimum glucose, mmol/L | 4.4 (4.2, 4.8) | 4.7 (4.4, 5.0) | -0.3 (-0.5, -0.07), *p* = 0.01 |
| Maximum glucose, mmol/L | 10.8 (10.3, 11.4) | 11.0 (10.5, 11.6) | -0.2 (-0.6, 0.2), *p* = 0.32 |
| AUC, mmol/L/hr | 5.0 (4.8, 5.4) | 5.2 (5.0, 5.5) | -0.2 (-0.3, -0.02), *p* =0.02 |
| LBGI | 1.8 (0.7, 2.9) | 1.5 (0.3, 2.7) | 0.3 (-0.3, 0.9), *p* = 0.31 |
| MAGE, mg/dL | 68.3 (61.9, 74.7) | 69.6 (62.6, 76.6) | -1.3 (-6.3, 3.6), *p* = 0.60 |
| MAGE, mmol/L | 3.8 (3.4, 4.2) | 3.9 (3.5, 4.3) | -0.1 (-0.4, 0.2), *p* = 0.60 |
| Nocturnal glucose, mg/dL | 117 (111, 123) | 120 (113, 127) | -3.30 (-8.5, 1.96) *p* = 0.22 |
| Nocturnal glucose, mmol/L | 6.49 (6.16, 6.82) | 6.67 (6.30, 7.04) | -0.18 (-0.47, 0.11) *p* = 0.22 |
| Min glucose night after, mg/dL | 94.7 (88.9, 101) | 99.7 (93.3, 106) | -4.97 (-9.53, -0.40) *p* = 0.03 |
| Min glucose night after, mmol/L | 5.26 (4.94, 5.58) | 5.54 (5.18, 5.89) | -0.28 (-0.53, -0.02) *p* = 0.03 |
| Time in target range, 70-140 mg/dL, % | 67.2 (59.6, 74.8) | 65.5 (57.6, 73.3) | 1.7 (-1.6, 5.1), *p* = 0.32 |
| Time in target range, 140-180 mg/dL, % | 18.3 (12.7, 23.9) | 18.8 (13.1, 24.5) | -0.5 (-2.8, 1.8), *p* = 0.69 |
| Time above target range, 180-250 mg/dL, % | 7.0 (4.0, 10.1) | 8.4 (5.1, 11.6) | -1.3 (-3.3, 0.5), *p* = 0.17 |
| Time above target range, >250 mg/dL, % | 2.14 (-0.8, 5.1) | 3.38 (0.4, 6.4) | -1.2 (-2.4, -0.1), *p* = 0.03 |
| Total number of hypoglycemic events | 0.4 (0.1, 0.7) | 0.5 (0.2, 0.8) | 0.06 (-0.1, 0.3), *p* = 0.54 |
| Number of level 2 hypoglycemic events | 0.2 (0.1, 0.3) | 0.1 (-0.1, 0.2) | 0.1 (0, 0.2), *p* = 0.07 |
| Number of prolonged hypoglycemic events | 0.05 (0, 0.1) | 0.01 (-0.1, 0.1) | 0.03 (-0.3, 0.1), *p* = 0.33 |
| Average length of hypoglycemic events, mins | 28.1 (5.68, 50.5) | 25.4 (1.14, 49.7) | 2.69 (-12.73, 18.39) *p* = 0.73 |
| Total number of hyperglycemic events | 1.2 (0.8, 1.6) | 1.1 (0.7, 1.5) | -0.1 (-0.4, 0.1), *p* = 0.29 |
| Number of level 2 hyperglycemic events | 0.1 (0.0, 0.2) | 0.1 (0.1, 0.2) | -0.03 (-0.1, 0.02), P= 0.31 |
| Number of prolonged hyperglycemic events | 0.05 (0.0, 0.10) | 0.05 (0.0, 0.11) | 0 (-0.04, 0.03), *p* = 0.69 |
| Average length of hyperglycemic events, mins | 70.9 (32.2, 110) | 91.2 (50.0, 132) | -20.27 (-44.77, 5.15) *p* = 0.11 |
| Total time spent in hyperglycemic events, mins | 59.3 (22.9, 95.6) | 65.2 (24.3, 106.1) | -5.96 (-36.92, 24.63) *p* = 0.70 |

Additional CGM metrics between exercise and non-exercise days at T1.

**Supplemental Table 5.** Additional CGM Metrics Between Exercise Types at T1.

| Metric | Moderate | Vigorous | Interval | Strength | Other | No Exercise | *p* Value |
| --- | --- | --- | --- | --- | --- | --- | --- |
| Mean 24-hour glucose, mmol/L | 6.88 (6.50, 7.26) | 6.92 (6.50, 7.35) | 7.05 (6.01, 8.09) | 7.13 (6.67, 7.58) | 6.83 (6.10, 7.56) | 7.16 (6.77, 7.54) | 0.13 |
| Mean 24-hour glucose, mg/dL | 124 (117, 131) | 125 (117, 132) | 127 (108, 146) | 128 (120, 136) | 123 (110, 136) | 129 (122, 136) | 0.13 |
| SD, mg/dL | 27.0 (24.0, 40.0) | 27.8 (24.2, 31.4) | 28.2 (18.4, 38.0) | 27.0 (23.1, 30.8) | 24.5 (17.9, 31.2) | 27.9 (24.8, 31.0) | 0.85 |
| SD, mmol/L | 1.50 (1.33, 2.67) | 1.54 (1.34, 1.74) | 1.57 (1.03, 2.11) | 1.50 (1.29, 1.71) | 1.36 (1.00, 1.74) | 1.55 (1.38, 1.72) | 0.85 |
| eA1c, % | 5.97 (5.73, 6.21) | 6.00 (5.73, 6.26) | 6.08 (5.42, 6.73) | 6.12 (5.84, 6.41) | 5.94 (5.48, 6.39) | 6.14 (5.90, 6.39) | 0.13 |
| Minimum glucose, mmol/L | 4.44 (4.16, 4.72) | 4.43 (4.08, 4.77) | 4.17 (3.13, 5.20) | 4.77 (4.39, 5.15) | 4.42 (3.72, 5.11) | 4.74 (4.45, 5.04) | 0.03 |
| Minimum glucose, mg/dL | 79.9 (74.9, 85.0) | 79.7 (73.4, 85.9) | 75.0 (56.4, 93.6) | 85.9 (79.1, 92.6) | 79.5 (67.0, 92.0) | 85.4 (80.1, 90.7) | 0.03 |
| Maximum glucose, mmol/L | 10.8 (10.29, 11.4) | 10.9 (10.17, 11.6) | 11.8 (9.64, 14.0) | 10.8 (9.99, 11.5) | 10.6 (9.09, 12.0) | 11.0 (10.46, 11.6) | 0.84 |
| Maximum glucose, mg/dL | 195 (185, 205) | 196 (183, 209) | 213 (174, 253) | 194 (180, 208) | 190 (164, 217) | 199 (188, 209) | 0.84 |
| AUC, mmol/L/hr | 5.03 (4.76, 5.31) | 5.15 (4.83, 5.46) | 5.27 (4.49, 6.05) | 5.22 (4.89, 5.56) | 4.93 (4.39, 5.47) | 5.24 (4.96, 5.52) | 0.11 |
| AUC, mg/dL/hr | 90.6 (85.7, 95.5) | 92.6 (87.0, 98.3) | 94.9 (80.9, 108.8) | 94.0 (88.1, 100.0) | 88.8 (79.0, 98.5) | 94.3 (89.2, 99.4) | 0.11 |
| Coefficient of variation, % | 21.8 (19.8, 23.8) | 21.7 (19.1, 24.2) | 21.9 (13.9, 29.9) | 20.5 (17.7, 23.3) | 18.9 (13.6, 24.3) | 21.5 (19.4, 23.6) | 0.83 |
| HBGI | 2.88 (1.32, 4.44) | 2.60 (0.94, 4.27) | 3.09 (-0.16, 6.34) | 3.40 (1.68, 5.12) | 2.44 (0.04, 4.83) | 3.52 (1.95, 5.10) | 0.21 |
| LBGI | 1.94 (0.81, 3.08) | 1.42 (0.13, 2.72) | 0.77 (-2.46, 4.00) | 1.63 (0.26, 3.00) | 1.30 (-0.95, 3.54) | 1.50 (0.34, 2.67) | 0.61 |
| MAGE, mg/dL | 68.1 (61.5, 74.7) | 71.0 (62.5, 79.5) | 73.8 (46.9, 100.7) | 66.3 (57.0, 75.7) | 57.9 (40.0, 75.9) | 69.5 (62.5, 76.4) | 0.72 |
| MAGE, mmol/L | 3.78 (3.42, 4.15) | 3.95 (3.47, 4.42) | 4.10 (2.60, 5.60) | 3.69 (3.17, 4.20) | 3.22 (2.22, 4.22) | 3.86 (3.47, 4.25) | 0.72 |
| Nocturnal glucose, mg/dL | 115 (108.7, 121) | 119 (110.7, 127) | 116 (87.6, 144) | 126 (116.5, 135) | 112 (93.8, 131) | 120 (113.6, 127) | 0.12 |
| Nocturnal glucose, mmol/L | 6.38 (6.04, 6.71) | 6.62 (6.15, 7.08) | 6.44 (4.87, 8.02) | 6.99 (6.47, 7.50) | 6.25 (5.21, 7.29) | 6.68 (6.31, 7.04) | 0.12 |
| Time in target range, 70-180 mg/dL, % | 85.6  (79.2, 92.0) | 87.3 (80.1, 94.5) | 87.3  (70.2, 104.5) | 82.4 (74.9, 90.0) | 91.4 (79.3, 92.0) | 84.3 (77.7, 90.8) | 0.49 |
| Time in target range, 70-140 mg/dL, % | 67.0 (59.3, 74.7) | 69.3 (60.8, 77.8) | 68.5 (49.6, 87.4) | 65.0 (56.1, 73.9) | 73.7 (60.3, 87.2) | 65.5  (57.6, 73.3) | 0.53 |
| Time above range, 140-180 mg/dL, % | 18.6 (12.94, 24.3) | 18.1 (11.91, 24.2) | 18.8 (5.71, 32.0) | 17.4 (11.00, 23.8) | 17.6 (8.12, 27.0) | 18.8 (13.06, 24.6) | 0.99 |
| Time above target range, 180-250 mg/dL, % | 6.44 (3.34, 9.55) | 7.18 (3.48, 10.88) | 7.31 (-2.95, 17.57) | 10.32 (6.34, 14.29) | 3.72 (-3.27, 10.71) | 8.37 (5.15, 11.60) | 0.11 |
| Number of level 2 hypoglycemic events | 0.23 (0.08, 0.37) | 0.07 (-0.11, 0.26) | -0.01 (-0.62, 0.59) | 0.16 (-0.05, 0.37) | 0.06 (-0.34, 0.47) | 0.09 (-0.07, 0.24) | 0.15 |
| Number of prolonged hypoglycemic events | 0.06 (0.01, 0.12) | 0.02 (-0.08, 0.11) | -0.05 (-0.44, 0.34) | 0 (-0.11, 0.11) | 0.02 (-0.23, 0.27) | 0.01 (-0.05, 0.08) | 0.71 |
| Total number of hyperglycemic events | 1.02 (0.63, 1.41) | 1.25 (0.79, 1.71) | 1.30 (0.01, 2.59) | 1.19 (0.69, 1.69) | 1.28 (0.40, 2.16) | 1.21 (0.81, 1.61) | 0.58 |
| Total number of hypoglycemic events | 0.54 (0.26, 0.82) | 0.33 (-0.03, 0.68) | 1.00 (-0.08, 2.09) | 0.34 (-0.04, 0.73) | 0.43 (-0.30, 1.15) | 0.42 (0.12, 0.71) | 0.49 |
| Number of level 2 hyperglycemic events | 0.11 (0.03, 0.20) | 0.11 (0.0, 0.22) | 0.11 (-0.22, 0.45) | 0.12 (0.01, 0.24) | 0.10 (-0.13, 0.32) | 0.14 (0.05, 0.23) | 0.95 |
| Number of prolonged hyperglycemic events | 0.04 (-0.01, 0.09) | 0.08 (0.02, 0.15) | 0.05 (-0.18, 0.27) | 0.04 (-0.03, 0.11) | 0.04 (-0.11, 0.19) | 0.06 (0.0, 0.11) | 0.85 |
| Total time spent in hyperglycemic events | 64.8 (26.74, 102.8) | 45.8 (-4.06, 95.6) | 25.8 (-111.37, 163) | 51.6 (-2.86, 106.1) | 49.8 (-54.2, 153.7) | 65.5 (24.51, 106.5) | 0.93 |
| Total time spent in hypoglycaemic events, mins | 78.4 (10.6, 146) | 50.0 (-24.4, 124) | 57.1 (-85.9, 200) | 66.9 (-10.5, 144) | 18.5 (-96.3, 133) | 57.5 (-11.7, 127) | 0.55 |

Additional CGM metrics between different exercise types at T1.

**Supplemental Table 6.** CGM Metrics at T2.

| Metric | Exercise Day | No Exercise Day | Between-Group Effect Estimate |
| --- | --- | --- | --- |
| Mean 24-hour glucose, mg/dL | 129 (116, 142) | 135 (122, 149) | -6 (-12, -1), *p* = 0.03 |
| Mean 24-hour glucose, mmol/L | 7.16 (6.43, 7.90) | 7.51 (6.77, 8.25) | -0.35 (-0.67, -0.04), *p* = 0.03 |
| Coefficient of variation, % | 19.3 (16.5, 22.1) | 18.2 (15.4, 21.1) | 1.0 (-1.0, 3.0), *p* = 0.30 |
| SD, mg/dL | 27.0 (21.6, 32.4) | 26.7 (21.2, 32.2) | 0.26 (-2.51, 2.87), *p* = 0.85 |
| SD, mmol/L | 1.50 (1.20, 1.80) | 1.48 (1.18, 1.79) | 0.01 (-0.14, 0.16), *p* = 0.85 |
| eA1c, % | 6.14 (5.68, 6.61) | 6.37 (5.90, 6.83) | -0.22 (-0.42, -0.03), *p* = 0.03 |
| Minimum glucose, mg/dL | 84.1 (73.8, 94.5) | 90.1 (79.5, 100.6) | -5.97 (-12, 0), *p* = 0.05 |
| Minimum glucose, mmol/L | 4.67 (4.10, 5.25) | 5.00 (4.42, 5.59) | -0.33 (-1, 0), *p* = 0.05 |
| Maximum glucose, mg/dL | 194 (174, 214) | 194 (174, 215) | -0.47 (-10.87, 9.32), *p* = 0.93 |
| Maximum glucose, mmol/L | 10.8 (9.65, 11.9) | 10.8 (9.66, 11.9) | -0.03 (-0.60, 0.52), *p* = 0.93 |
| AUC, mg/dL/hr | 94.1 (84.7, 104) | 98.5 (89.0, 108) | -4.4 (-8.9, 0), *p* = 0.05 |
| AUC, mmol/L/hr | 5.23 (4.71, 5.75) | 5.47 (4.95, 6.00) | -0.24 (-0.5, 0), *p* = 0.05 |
| LBGI | 1.67 (0.76, 2.57) | 1.08 (0.04, 2.11) | 0.59 (-0.51, 1.70), *p* = 0.30 |
| HBGI | 4.50 (1.60, 7.40) | 5.60 (2.68, 8.51) | -1.10 (-2.00, -0.18), *p* = 0.02 |
| MAGE, mg/dL | 68.8 (53.4, 84.2) | 70.6 (54.9, 86.3) | -1.80 (-11.33, 7.00), *p* = 0.70 |
| MAGE, mmol/L | 3.82 (2.97, 4.68) | 3.92 (3.05, 4.80) | -0.10 (-0.63, 0.39), *p* = 0.70 |
| Nocturnal glucose, mg/dL | 118 (102, 133) | 125 (110, 141) | -7.30 (-15.77, 1.34), *p* =0.09 |
| Nocturnal glucose, mmol/L | 6.55 (5.69, 7.40) | 6.95 (6.08, 7.82) | -0.41 (-0.88, 0.07), *p* =0.09 |
| Time in target range, 70-180 mg/dL, % | 87.0 (76.0, 97.9) | 88.1 (77.1, 99.1) | -1.15 (-6.58, 4.13), *p* = 0.67 |
| Time in target range, 70-140 mg/dL, % | 79.2 (66.1, 92.3) | 78.0 (64.7, 91.2) | 1.23 (-3.92, 6.40), *p* = 0.64 |
| Time in target range, 140-180 mg/dL, % | 7.71 (-0.78, 16.2) | 10.10 (1.51, 18.70) | -2.40 (-6.57, 1.63), *p* = 0.26 |
| Time above target range, 180-250 mg/dL, % | 2.49 (-6.25, 11.2) | 1.68 (-7.13, 10.5) | 0.81 (-2.68, 4.34), *p* = 0.65 |
| Total number of hypoglycemic events | 0.51 (0.13, 0.88) | 0.43 (0.04, 0.81) | 0.08 (-0.22, 0.36), *p* = 0.60 |
| Number of level 2 hypoglycemic events | 0.08 (0.0, 0.16) | 0.0 (-0.08, 0.09) | 0.07 (0.0, 0.14), *p* = 0.05 |
| Number of prolonged hypoglycemic events | 0.02 (0.0, 0.04) | 0.0 (-0.02, 0.03) | 0.02 (-0.02, 0.04), *p* = 0.37 |
| Total number of hyperglycemic events | 0.39 (-0.23, 1.01) | 0.27 (-0.36, 0.90) | 0.11 (-0.25, 0.45), *p* = 0.53 |
| Number of level 2 hyperglycemic events | 0.07 (-0.04, 0.18) | 0.11 (0.0, 0.23) | -0.04 (-0.11, 0.03), *p* = 0.20 |
| Number of prolonged hyperglycemic events | 0.09 (0.02, 0.16) | 0.09 (0.03, 0.16) | 0.0 (-0.05, 0.05), *p* = 0.89 |

CGM metrics between exercise and non-exercise days at T2.

**Supplemental Table 7.** Additional CGM Metrics Between Exercise Types at T2.

| Metric | Moderate | Vigorous | Interval | Strength | Other | No Exercise | *p* Value |
| --- | --- | --- | --- | --- | --- | --- | --- |
| Mean 24-hour glucose, mg/dL | 131 (117.6, 145) | 126 (111.6, 140) | 120 (89.9, 151) | 118 (96.9, 139) | 129 (104.0, 153) | 135 (121.8, 149) | 0.08 |
| Mean 24-hour glucose, mmol/L | 7.29 (6.53, 8.04) | 6.98 (6.20, 7.76) | 6.69 (5.00, 8.39) | 6.57 (5.39, 7.75) | 7.14 (5.78, 8.50) | 7.52 (6.77, 8.27) | 0.08 |
| Coefficient of variation, % | 19.2 (16.3, 22.1) | 19.0 (15.9, 22.2) | 26.3 (15.9, 36.7) | 19.1 (12.5, 25.8) | 15.7 (7.7, 23.7) | 18.2 (15.3, 21.1) | 0.58 |
| SD, mg/dL | 27.1 (21.6, 32.6) | 26.5 (20.8, 32.3) | 30.9 (16.4, 45.3) | 27.4 (17.7, 37.1) | 21.9 (10.5, 33.3) | 26.7 (21.2, 32.2) | 0.91 |
| SD, mmol/L | 1.51 (1.20, 1.81) | 1.47 (1.15, 1.80) | 1.72 (0.92, 2.52) | 1.52 (0.98, 2.06) | 1.22 (0.58, 1.85) | 1.48 (1.18, 1.79) | 0.91 |
| eA1c, % | 6.22 (5.75, 6.70) | 6.03 (5.54, 6.52) | 5.86 (4.79, 6.93) | 5.77 (5.02, 6.51) | 6.13 (5.27, 6.99) | 6.37 (5.90, 6.84) | 0.08 |
| Minimum glucose, mg/dL | 84.6 (74.3, 94.8) | 83.6 (72.6, 94.6) | 79.1 (46.8, 111.5) | 78.2 (57.1, 99.4) | 104.8 (79.7, 130.0) | 90.2 (80.0, 100.5) | 0.19 |
| Minimum glucose, mmol/L | 4.70 (4.13, 5.27) | 4.64 (4.03, 5.25) | 4.40 (2.60, 6.19) | 4.35 (3.17, 5.52) | 5.82 (4.43, 7.22) | 5.01 (4.45, 5.58) | 0.19 |
| Maximum glucose, mg/dL | 193 (172, 214) | 196 (174, 217) | 207 (153, 261) | 186 (150, 223) | 189 (146, 232) | 194 (173, 214) | 0.98 |
| Maximum glucose, mmol/L | 10.7 (9.57, 11.9) | 10.9 (9.66, 12.1) | 11.5 (8.47, 14.5) | 10.4 (8.32, 12.4) | 10.5 (8.12, 12.9) | 10.8 (9.62, 11.9) | 0.98 |
| AUC, mg/dL/hr | 95.8 (86.1, 105) | 91.9 (81.9, 102) | 86.6 (62.8, 110) | 86.1 (69.8, 102) | 93.8 (74.9, 113) | 98.6 (88.9, 108) | 0.16 |
| AUC, mmol/L/hr | 5.32 (4.78, 5.86) | 5.11 (4.55, 5.67) | 4.81 (3.49, 6.13) | 4.78 (3.88, 5.69) | 5.21 (4.16, 6.26) | 5.48 (4.94, 6.01) | 0.16 |
| LBGI | 1.14 (0.11, 2.18) | 2.51 (1.24, 3.77) | 3.55 (-2.60, 9.70) | 1.10 (-2.62, 4.82) | 1.10 (-3.41, 5.60) | 0.98 (-0.10, 2.05) | 0.41 |
| HBGI | 4.85 (1.90, 7.80) | 3.94 (0.94, 6.94) | 4.98 (-0.25, 10.21) | 2.16 (-1.75, 6.07) | 3.79 (-0.57, 8.15) | 5.58 (2.63, 8.53) | 0.04 |
| MAGE, mg/dL | 69.0 (53.39, 84.6) | 68.9 (52.20, 85.6) | 74.5 (26.02, 123.1) | 63.8 (32.02, 95.6) | 45.5 (7.73, 83.2) | 70.2 (54.58, 85.8) | 0.85 |
| MAGE, mmol/L | 3.83 (2.97, 4.70) | 3.83 (2.90, 4.76) | 4.14 (1.45, 6.84) | 3.54 (1.78, 5.31) | 2.53 (0.43, 4.62) | 3.90 (3.03, 4.76) | 0.85 |
| Nocturnal glucose, mg/dL | 121 (104.9, 137) | 112 (94.6, 129) | 121 (75.5, 167) | 114 (83.6, 144) | 100 (64.6, 135) | 125 (109.3, 142) | 0.19 |
| Nocturnal glucose, mmol/L | 6.73 (5.83, 7.63) | 6.21 (5.25, 7.16) | 6.72 (4.20, 9.25) | 6.32 (4.65, 7.99) | 5.56 (3.59, 7.52) | 6.97 (6.07, 7.87) | 0.19 |
| Time in target range, 70-180 mg/dL, % | 88.4 (77.1, 99.8) | 84.1 (72.3, 96.0) | 60.7 (32.8, 88.6) | 106.5 (87.4, 125.5) | 84.9 (62.8, 107.1) | 89.1 (77.7, 100.5) | 0.04 |
| Time in target range, 70-140 mg/dL, % | 81.1 (67.7, 94.4) | 76.8 (63.1, 90.5) | 50.5 (22.4, 78.5) | 84.1 (64.3, 104.0) | 75.7 (53.0, 98.4) | 78.5 (65.2, 91.8) | 0.17 |
| Time in target range, 140-180 mg/dL, % | 7.34 (-1.34, 16.0) | 7.13 (-1.93, 16.2) | 10.20 (-11.61, 32.0) | 22.87 (8.05, 37.7) | 10.41 (-6.88, 27.7) | 10.61 (1.94, 19.3) | 0.21 |
| Time above target range, 180-250 mg/dL, % | 2.65 (-6.21, 11.51)) | 3.07 (-6.04, 12.18) | 2.01 (-16.66, 20.68) | -12.12 (-25.34, 1.11) | 13.85 (-1.28, 28.97) | 1.34 (-7.52, 10.19) | 0.02 |
| Total number of hypoglycemic events | 0.41 (0.09, 0.73) | 0.43 (0.07, 0.79) | 5.13 (3.78, 6.48) | 0.38 (-0.47, 1.23) | 0.39 (-0.63, 1.42) | 0.40 (0.08, 0.72) | 0.68 |
| Number of level 2 hypoglycemic events | 0.08 (0, 0.17) | 0.08 (-0.02, 0.17) | 0 (-0.39, 0.39) | 0.03 (-0.21, 0.28) | 0.04 (-0.25, 0.34) | 0.0 (-0.08, 0.09) | 0.53 |
| Number of prolonged hypoglycemic events | 0.0 (-0.02, 0.03) | 0.04 (0.01, 0.08) | -0.03 (-0.19, 0.14) | 0.01 (-0.09, 0.11) | 0.01 (-0.11, 0.13) | 0.0 (-0.03, 0.03) | 0.36 |
| Total number of hyperglycemic events | 0.44 (-0.22, 1.10) | 0.38 (-0.32, 1.07) | 0.20 (-1.64, 2.05) | -0.36 (-1.59, 0.87) | -0.64 (-2.09, 0.81) | 0.24 (-0.42, 0.90) | 0.50 |
| Number of level 2 hyperglycemic events | 0.06 (-0.05, 0.17) | 0.06 (-0.05, 0.18) | 0.09 (-0.25, 0.43) | 0.45 (0.23, 0.67) | 0.10 (-0.16, 0.36) | 0.13 (0.02, 0.23) | 0.01 |
| Number of prolonged hyperglycemic events | 0.10 (0.03, 0.17) | 0.09 (0.0, 0.16) | 0.1 (-0.16, 0.35) | -0.01 (-0.18, 0.15) | -0.05 (-0.25, 0.14) | 0.09 (0.02, 0.16) | 0.54 |

CGM metrics between different exercise types at T2.

**Supplemental Table 8.** Exercise Session Analysis Breakdown at the 24-week Time Point (T1) and at 50-52-week Follow-up (T2)

|  | **T1** | | | **T2** | | |
| --- | --- | --- | --- | --- | --- | --- |
| **Type of exercise session** | **n (%)** | **Heart rate, bpm, mean (SD)** | **Duration, mins, mean (SD)** | **n (%)** | **Heart rate, bpm, mean (SD)** | **Duration, mins, mean (SD)** |
| **Overall** | **300** | **-** | **-** | **105** | **-** | **-** |
| Moderate, n (%) | 211 (70) | 108 (13) | 55 (59) | 64 (61) | 106 (12) | 67 (50) |
| Vigorous, n (%) | 48 (16) | 121 (9) | 45 (25) | 32 (30) | 122 (12) | 38 (17) |
| Interval, n (%) | 3 (1) | 124 (16) | 33 (45) | 1 (1) | 95 (N/A) | 120 |
| Strength, n (%) | 32 (11) | 103 (15) | 44 (113) | 6 (6) | 110 (10) | 59 (13) |
| Other, n (%) | 6 (2) | 94 (11) | 45 (15) | 2 (2) | 102 (2) | 54 (11) |
| **Non-exercise days** | 106 | - | - | 53 | - | - |

Descriptive statistics of the type of exercise sessions and non-exercise days.

**SUPPLEMENTAL METHODS**

**Recruitment**

Recruitment for the trial took place over 13 months, starting in January 2021, in the UK and Canada. In the UK, participants were recruited through GP database searches, as well as through clinical staff at participating GP practices in various cities. Recruitment also included flyers at diabetes education sessions, posters in healthcare facilities, and promotion via social media and diabetes websites. In Canada, recruitment strategies involved advertisements on the Diabetes Canada website, employing online recruitment services, local online classifieds, print media, and physical activity initiatives. Additional recruitment efforts included distributing posters and invitation letters to pharmacies and general practitioners.

**Eligibility Criteria**

Eligibility criteria included: participants who had a recent clinical diagnosis of type 2 diabetes (within the previous 5-24 months), were aged between 40-75 years, and who managed their type 2 diabetes by lifestyle modifications alone or metformin (stable dose for ≥3 months). Participants were excluded from the study if they were under 40 or over 75 years, had an HbA1c greater than 10% (>86mmol/mol), or had blood pressure exceeding 160/100 mm Hg. Additional exclusion criteria included the use of oral antidiabetic drugs other than metformin, insulin use, unstable angina, myocardial infarction within the past 3 months, transient ischemic attack (TIA) within the past 6 months, heart failure of class II or higher, arrhythmia, or an inability to increase activity levels. Pregnant individuals or those planning pregnancy, those less than 6 months postpartum or who stopped breastfeeding within the last month, and individuals without a smartphone or data/Wi-Fi access were also excluded. Lastly, participants who were already meeting the recommended exercise guidelines of 150 minutes of moderate-intensity exercise per week were excluded.

**Exercise Session Analysis**

Due to the free-living, unsupervised, individualized nature of the study, sessions were assigned according to the predominant HR zone and pattern achieved during the session and in conjunction with what was known to be prescribed by the exercise specialist for the session, along with what exercise type was selected by the participant on the fitness watch. Moderate aerobic sessions were defined as at least 50% of the duration of a session where HR was 60-70% of age predicted HRmax. Vigorous aerobic sessions were defined as 50% of the duration of a session where HR was >70% age predicted HRmax and where no evidence of an interval pattern (e.g., intermittent HR decline to below 60% HRmax) were present. Interval training sessions were defined as sessions where at least 2 or more intervals (regardless of interval time) were noted, where >70% HRmax was achieved at some point during the interval, and where recovery (HR decline) between intervals was present. Strength training sessions were defined through comparing session prescription/participant-recorded session notes and text message feedback whenever the participant marked a session as strength. Sessions were classified as ‘other’ when type was not noted by the participant and the HR did not ever exceed 50% of age predicated HRmax at any time during the session.

**Statistical Analysis**

The linear mixed model included fixed effects for condition, a random effect for participant ID to account for repeated measures, and adjustments for age, sex, and baseline HbA1c. Assumptions were assessed via visual inspection of diagnostic plots. Estimated marginal means derived from the model are presented for each condition. Preplanned between-condition comparisons are reported as the main effect of interest and presented as effect estimates alongside 95% confidence limits. The same model was used to assess differences across secondary outcomes (CGM metrics according to the CGM consensus statement). No missing data were imputed as per contemporary guidelines for linear mixed models with longitudinal data (1). Statistical tests were performed using R (version 4.3.2, RStudio, PBC, Boston, MA). Statistical significance was accepted when P < 0.05.

**SUPPLEMENTAL RESULTS**

**Study Participants**

At T2, 18 participants (33% female) had sufficient data in the 14-day testing period. Mean (±SD) CGM wear-time was 12.6 ± 2.4 days at T1 and 12.8 ± 1.8 at T2. Additional participant characteristics at T2 can be found in Supplemental Table 1.

**Exercise Session Timing**

Of the 405 exercise sessions, 40% of the exercise sessions were completed in the evening (18:00-0:00 hrs), 36% were completed in the afternoon (12:00-18:00 hrs), and 24% were completed in the morning (0:00-12:00). 63% of the exercise sessions were completed on a weekday (Monday-Thursday) and 37% were completed on a weekend day (Friday-Sunday).

**Exercise Types**

Results for the differences in exercise types for the T2 time point were similar to the T1 time point (see Supplementary Table 6).

**REFERENCES FOR SUPPLEMENTAL MATERIAL**

1. Chakraborty H. A mixed model approach for intent-to-treat analysis in longitudinal clinical trials with missing values [Internet]. Research Triangle Park, NC; 2009 Apr. Available from: http://www.rti.org/publication/mixed-model-approach-intent-treat-analysis-longitudinal-clinical-trials-missing-values
